# ABCA1 is associated with the development of acquired chemotherapy resistance and predicts poor ovarian cancer outcome

**DOI:** 10.20517/cdr.2020.107

**Published:** 2021-06-19

**Authors:** Wanqi Wang, Noor A Lokman, Tannith M Noye, Anne M Macpherson, Martin K Oehler, Carmela Ricciardelli

**Affiliations:** ^1^Adelaide Medical School, Robinson Research Institute, University of Adelaide, Adelaide, SA 5000, Australia.; ^2^Department of Gynaecological Oncology, Royal Adelaide Hospital, Adelaide, SA 5000, Australia.

**Keywords:** HGSOC, chemotherapy resistance, ABC transporter, *ABCA1*, *ABCB1*, *TAP2*, *ABCB3*, *ABCC2*, *ABCG2*, apabetalone

## Abstract

**Aim**: This study investigated the ATP binding cassette (ABC) transporter (ABCA1, ABCB1, ABCB3, ABCC2 and ABCG2) expression in high grade serous ovarian cancer (HGSOC) tissues, cell lines and primary cells to determine their potential relationship with acquired chemotherapy resistance and patient outcome.

**Methods**: ABC transporter mRNA and protein expression (ABCA1, ABCB1, ABCB3, ABCC2 and ABCG2) was assessed in publicly available datasets and in a tissue microarray (TMA) cohort of HGSOC at diagnosis, respectively. ABC transporter mRNA expression was also assessed in chemosensitive ovarian cancer cell lines (OVCAR-5 and CaOV3) versus matching cell lines with acquired carboplatin resistance and in primary HGSOC cells from patients with chemosensitive disease at diagnosis (*n* = 10) as well as patients with acquired chemotherapy resistance at relapse (*n* = 6). The effects of the ABCA1 inhibitor apabetalone in carboplatin-sensitive and -resistant cell lines were also investigated.

**Results**: High *ABCA1* mRNA and protein expression was found to be significantly associated with poor patient outcome. *ABCA1* mRNA and protein levels were significantly increased in ovarian cancer cell lines (OVCAR-5 CBPR and CaOV3 CBPR) with acquired carboplatin resistance. *ABCA1* mRNA was significantly increased in primary HGSOC cells obtained from patients with acquired chemotherapy resistance. Apabetalone treatment reduced ABCA1 protein expression and increased the sensitivity of both parental and carboplatin-resistant ovarian cancer cells to carboplatin.

**Conclusion**: These results suggest that inhibiting ABCA1 transporter may be useful in overcoming acquired chemotherapy resistance and improving outcome for patients with HGSOC.

## Introduction

Ovarian cancer is the most lethal gynecological malignancy in the developed world^[1]^. Epithelial ovarian cancer, which includes serous, clear cell, mucinous and endometrioid subtypes, constitutes 90% of ovarian cancers^[2]^. Up to 70% of epithelial ovarian cancers are high grade serous ovarian cancers (HGSOC), which are the most common and deadliest ovarian cancer subtype^[2]^. HGSOC is usually diagnosed at an advanced stage and current treatment strategies include a combination of radical debulking surgery and chemotherapy (carboplatin + paclitaxel). Although the initial responses to chemotherapy treatment are high, up to 60% of ovarian cancer patients relapse within six months and 75% of patients ultimately become chemoresistant, which is the main factor contributing to ovarian cancer death^[2]^. The development of more effective therapies for chemotherapy disease is urgently required for improving the survival rate of ovarian cancer patients.

The basis for the chemoresistance is multifactorial involving both tumor and drug related factors^[3-6]^. A potential mechanism involves the increased expression of ATP-binding cassette (ABC) transporter membrane proteins, which can decrease levels of chemotherapy drugs within cells^[7]^. Although studies including ovarian cancer subtypes have suggested that ABC transporters are associated with reduced survival and chemotherapy resistance, the findings have been very inconsistent^[7,8]^.

ABC transporters constitute a ubiquitous superfamily of integral membrane proteins that are responsible for the ATP powered translocation of substrates across membranes. There are seven subfamilies of ABC transporters (ABCA-ABCG) including 49 ABC transporters in humans^[7]^. Most ABC transporters are inward opening and couple the hydrolysis of ATP to export molecules in a unidirectional path across the phospholipid bilayer of cellular membranes, against a chemical gradient^[7]^. Four subfamilies (ABCA ABCB, ABCC and ABCG) have been shown to be associated with drug resistance in ovarian cancer^[7]^.

This study investigated the relationship of the five ABC transporters ABCA1, ABCB1, ABCB3 (also known as TAP2), ABCC2 and ABCG2 with ovarian cancer chemoresistance and outcome. The rationale for selecting these ABC transporters were: (1) ABCA1 has been widely studied as a cholesterol transporter, and platinum drugs commonly used for ovarian cancer are drug substrates for ABCA1^[9]^. Increased ABCA1 mRNA/protein expression was found to be associated with poor clinical outcome in bowel^[10]^ and ovarian cancer^[11]^. It was also associated with lymph node metastasis in breast cancer^[12]^; (2) Paclitaxel that is commonly used for ovarian cancer patients is a drug substrate for ABCB1. Studies have shown that high ABCB1 mRNA/protein expression was associated with poor clinical outcome and chemoresistance in many cancers, including ovarian cancer^[13-16]^; However, some studies found no or the opposite relationship^[17,18]^; (3) ABCB3 was chosen as it is in the same subfamily as ABCB1 and closest in structure to ABCB1 but has not been widely studied in ovarian cancer^[7]^. Limited studies to date have shown that high ABCB3 mRNA/protein expression was associated with chemoresistance^[7,19]^ but better clinical outcome in ovarian cancer^[20,21]^; (4) Several studies have found ABCC2 overexpression in ovarian cancer patients with poor clinical outcome and chemotherapy response^[22,23]^, yet contrary studies exist^[24,25]^; and (5) ABCG2 is known as a stem cell marker that is associated with chemoresistance^[26,27]^ but has been poorly studied in ovarian cancer.

## Methods

### Analysis of public ovarian cancer microarray databases

Progression-free survival (PFS), post-progression survival (PPS) and overall survival (OS) Kaplan-Meir analyses were performed using Affymetrix mRNA microarray expression data from the Kaplan-Meier plotter (http://kmplot.com/analysis/index.php?p=service&cancer=ovar)^[28]^. Gene probes included *ABCA1* (203504_s_at, 203505_at and 216066_at), *ABCB1* (209993_at and 209994_s_at), *ABCB3/TAP2* (204769_s_at, 204770_at, 225973_at and 208428_at), *ABCC2* (206155_at) and *ABCG2* (209735_at). The online plotter tool was used to select the best cut-off to split the patients into two groups (high and low expression) for each analysis. For transporters with more than one mRNA probe, the mean expression was calculated by the online plotter tool. The analyses were performed on the 2017 version of 13 public microarray databases for all ovarian cancer patients (serous and endometroid) or HGSOC (serous grade 2 and 3) patients^[28]^.

### Patient tissue cohort

Tissue microarrays (TMA) (1 mm diameter tissue cores) in triplicate were obtained from a uniform cohort of HGSOC (*n* = 147) diagnosed between 1988 and 2013. Supplement Table 1 summarizes the clinicopathological characteristics of the TMA patient cohort. The research was conducted with patient consent and approval by the Royal Adelaide Hospital Human Ethics Committee (RAH protocols #060903 and #140201). Up to 81.5% of the patients received platinum chemotherapy as first line treatment.

### Immunohistochemistry

Immunohistochemistry was performed on TMA and tissue sections as described previously^[29]^. Archived formalin fixed paraffin embedded tissue sections (5 μm) were incubated at 60 °C (1.5 h), dewaxed and rehydrated using xylene and decreasing concentrations of ethanol. Tissue sections were washed with PBS and blocked for endogenous peroxidase activity with 0.3% hydrogen peroxide in PBS (5 min). Sections then underwent steam microwave antigen retrieval in 10 mM citric acid buffer, pH 6 at 100 °C (10 min) (Sixth sense, Whirlpool, Australia). TMAs were blocked with 5% goat serum (30 min) and incubated overnight at 4 °C with primary antibodies: ABCA1 (1/200, polyclonal, NB400-105, Novus Bio)^[30,31]^, ABCB1 (1/1200, clone F4, P7965, Sigma Aldrich)^[32]^, ABCB3 (1/750, TAP2, Ab130414, Abcam Cambridge UK)^[7]^, ABCC2 (1/50, clone M2I-4, Ab3372, Abcam)^[33]^ and ABCG2 (1/100, clone BXP-21, Abcam)^[34]^. The next day, sections were incubated sequentially with secondary antibodies: biotinylated goat anti-rabbit (1/400, Dako, Australia) for ABCA1 and ABCB3 or biotinylated goat anti-mouse (1/400, Dako, Australia) for ABCB1, ABCC2 and ABCG2, followed by streptavidin-horseradish peroxidase (1/500, Dako, Australia) at room temperature (1 h). Peroxidase activity was detected using diaminobenzidine (DAB) and H_2_O_2_ (Sigma-Aldrich). Sections were counterstained with hematoxylin (Sigma-Aldrich), dehydrated with 70% and 100% ethanol and xylene, and then mounted in Pertex (Medite Medizintechnik, Germany). Tissues without primary antibody or mouse/rabbit immunoglobulins were included as negative controls. Previous studies have found ABCA1^[35]^, ABCB1^[36,37]^ and ABCB3^[38]^ expression in colon tissue and ABCC2^[37,39]^ and ABCG2^[37]^ expression in liver tissue, and these were used as positive controls for the immunohistochemistry.

### Immunohistochemistry assessment

Tissue sections were scanned by NanoZoomer Digital Pathology System (Hamamatsu Photonics, SZK, Japan) and viewed by NDP view imaging software (NDP scan software v2.3, Hamamatsu Photonics). Intensity levels of ABC transporters in serous ovarian cancer cells and the percentage of positively stained cells were assessed using a manual scoring method by three independent researchers as described previously^[40]^. Staining intensity was graded as 0: negative; 1: weak; 2: moderate; or 3: strong. The percentage of positively stained tumor cells was scored as 0: none; 1: ≤ 10% positive cells; 2: 11%-50% positive cells; 3: 51%-80% positive cells; or 4: > 80% positive cells. Immunoreactive score (IR) was calculated by multiplying the percentage of positive cells with the intensity of staining^[40]^.

### Cell culture

Human serous ovarian cancer cell line CaOV3 was purchased from American Type Culture Collection. OVCAR-5 cells were obtained from Dr Thomas Hamilton (Fox Chase Cancer Center, PA, USA). Cell lines were grown in RPMI-1640 media (Sigma Aldrich, catalog number R8758, OVCAR-5) or DMEM media (Gibco, Life Technologies, catalog number 10567-022, CaOV3), cultured at 37 °C in a humidified environment of 5% CO_2_, with 10% fetal bovine serum (FBS, Scientifix, catalog number AFBS-500), antibiotics penicillin-streptomycin (1:100, Sigma-Aldrich, catalog number P4458) and antibiotic antimycotic solution (1:500, Sigma-Aldrich, catalog number A5955). OVCAR-5 and CaOV3 cells were made resistant to carboplatin (OVCAR-5 CBPR; CaOV3 CBPR) following treatment with eight cycles of carboplatin (CBP, 50 μM, Hospira Australia Pty, Ltd) as previously described^[29]^. The OVCAR-5 and CaOV3 carboplatin resistant (CBPR) cells exhibit a carboplatin IC_50_ that was nearly three-fold higher than that of the parental OVCAR-5 or CaOV3 cells [Supplementary Figure 1].

Primary serous ovarian cancer cells were derived from ascites collected from patients with chemosensitive disease at diagnosis (*n* = 9) or following the development of acquired chemoresistant disease (*n* = 6) as described previously^[41]^. Ascites was obtained with patient consent and approval by the Royal Adelaide Hospital (RAH protocol number 140201) and Central Adelaide Local Health Network Human Ethics Committees (CALHN #R20181215). Pathological and clinical characteristics of the patients whose ascites was used to isolate the primary cells are summarized in Supplement Table 2. All primary cells were grown in Advanced RPMI-1640 medium (Life Technologies catalog number 12633-020) supplemented with 2 mM GlutaMAX™ (Life Technologies, catalog number 35050061), 10% FBS and antibiotics between passages 1 and 4.

### Quantitative real-time PCR (qRT-PCR)

OVCAR-5, OVCAR-5 CBPR, CaOV-3 and CaOV-3 CBPR cell lines as well as primary ovarian cancer cells were plated at 5000 cells/well in 96-well plates and cultured for 72-96 h until confluent. Total RNA was isolated and reverse-transcribed using the TaqMan® Gene expression Cells-to CT kit (Applied Biosystems, Thermo Fisher Scientific, Waltham, MA, USA), as per the manufacturer’s instructions as previously described^[41]^. Briefly, lysis solution with DNAse was added to each well with incubation at room temperature (5 min). Then, stop solution was added and mixed to each well. Ten microliters of lysate were added to a 40 µL reverse transcription master mix for 1 h. Resultant cDNA was stored for qRT-PCR analysis as 50 µL aliquots at -20 °C. Quantstudio 12K Flex Real-Time PCR System (Applied Biosystems) was used for qRT-PCR reactions that were performed on triplicate samples using TaqMan® primer sets for *ABCA1* (Hs01059137), *ABCB1* (Hs00184500), *ABCB3* (Hs00241060), *ABCC2* (Hs00166123) and *ABCG2* (Hs01053790). PCR reactions were made up to 10 µL containing: TaqMan® Gene Expression Master Mix (2×), primers for the gene of interest, nuclease-free water and 2 µL sample cDNA. PCR cycling conditions were: 50 °C (2 min), 95 °C (10 min) followed by 40 cycles of 95 °C (15 s) and 60 °C (1 min). qRT-PCR negative controls included samples without RNA or cDNA. CT values were normalized to the housekeeping gene β-actin (Human ACTB 4333762, Applied Biosystems) and calibrator using the 2^-ΔΔCT^ method.

### Immunocytochemistry

Ovarian cancer cells (OVCAR-5, OVCAR-5 CBPR, CaOV3 and CaOV3 CBPR) were plated at 1 × 10^5^ cells/well in eight-well tissue culture chamber slides (Nunclon™ Lab-Tek II Chamber slide, ThermoFisher Scientific) in growth media (500 µL 10% FBS RPMI). Cells were fixed with ice-cold 100% methanol (3 min) and ice-cold 100% acetone (1 min), washed with PBS, blocked with 5% goat serum and incubated overnight with ABCA1 rabbit polyclonal antibody (1/100, NB400-105, Novus Biological). Protein expression was visualized with goat anti-rabbit Alexa Fluor® 488 for 1 h at room temperature (1/200, catalog number A11034, Molecular Probes, Life Technologies), and slides were mounted with Prolong Gold Antifade Mountant with DAPI (catalog number P36941, Molecular Probes, Life Technologies). Cells were viewed with an epifluorescence microscope (BX50, Olympus Australia) and imaged using a 40× objective and a Spot RT digital camera (Diagnostic Instruments, Sterling Heights, MI). Negative controls included rabbit immunoglobulin or no primary antibody.

### Cell survival assay

OVCAR-5 (5000 cells/well) and CaOV3 (7500 cells/well) cells were plated in 96-well plates in respective growth media. After 24 h, cells were treated with control media (DMSO, 0.06%), apabetalone (1-80 µM, RVX-208, catalog number S7295, SelleckChem, Houston, TX, USA), carboplatin (5-200 µM, Hospira, Australia) or apabetalone (80 µM) + carboplatin (5-200 µM) for 72 h. Cell survival was assessed by MTT assay as per the manufacturer’s instructions (Sigma Aldrich)^[42]^. Curve fitting using log(inhibitor) *vs.* normalized response - variable slope (Graph Pad Prism, Prism®, version 8.0.0, CA, USA) was used to calculate the carboplatin IC_50_ in the absence and presence of apabetalone. Combination index was determined according to the Chou-Talalay method^[43]^ using CompuSyn software (ComboSyn, Inc. New Jersey, USA). Drug interactions were considered synergistic, additive or antagonistic with combination index values of < 1, 1 and > 1, respectively.

### Western blotting

Protein extracts from ovarian cancer cell lines following 48-72 h treatment with apabetalone (20-80 µM prepared in RIPA buffer and electrophoresed on 4%-20% TGX gels (Bio-Rad Laboratories, Hercules, US) and transferred onto polyvinylidene difluoride membranes (GE Healthcare, Little Chalfont, England)^[29,44]^. The membranes were subsequently incubated with rabbit polyclonal antibody ABCA1 (1/1000, NB400-105, Novus Biological) for 2 h at room temperature and then peroxidase-conjugated anti-rabbit IgG (1/4000, Millipore, Australia) for 1 h at room temperature. Chemiluminescence (ECL Hyperfilm, GE Healthcare) was used to visualize protein expression. Membranes were scanned using ChemiDoc™ MP Imaging System (Bio-Rad Laboratories, Inc) and analyzed using Image Lab™ software (Version 6.0.1 build 34, Bio-Rad Laboratories, Inc). β-actin anti-rabbit antibody (1/5000, Abcam catalog number Ab8227) was used as a loading control.

### Statistical analyses

*ABCA1*, *ABCB1*, *ABCB3*, *ABCC2* and *ABCG2* mRNA expression in public datasets was analyzed by Kaplan-Meier online plotter and used to calculate the hazard ratio, 95%CI, log-rank *P* value and Kaplan-Meier survival curves^[28]^. In Kaplan-Meier online plotter, PPS is calculated from time of first progression to time of death, PFS survival is calculated from date of diagnosis to first progression and OS is calculated from date of diagnosis to date of death. Kaplan-Meier analyses were performed to assess the relationship of ABCA1, ABCB1, ABCB3, ABCC2 and ABCG2 protein expression in the HGSOC TMA cohort with PFS and OS (SPSS software, version 21.0, SPSS Inc., Chicago, IL, USA). Median IR scores or cut-off points either side of the median were used for Kaplan-Meir survival analysis. Relapse or death due to ovarian cancer was used as the endpoint. The following statistical analyses were all performed using Graph Pad Prism (Prism®, version 8.0.0): unpaired Student’s *t*-test for comparing mRNA expression between parental and carboplatin resistant cell lines, one-way ANOVA (Tukey’s multiple comparisons test) for assessing response to different concentrations of apabetalone and the Mann-Whitney *U* test for analyzing mRNA expression in chemosensitive and -resistant primary HGSOC cells. Statistical significance was accepted at *P* < 0.05.

## Results

### Relationship between ABC transporter mRNA expression with outcome in all ovarian cancer subtypes and HGSOC

By analyzing publicly available microarray data using the Kaplan-Meir online plotter^[28]^, high *ABCA1* and *ABCB3* mRNA expressions were significantly associated with reduced PFS in all ovarian cancer subtypes [Table 1A]. High ABCC2 and ABCG2 expressions were associated with increased PFS in all ovarian cancer subtypes [Table 1A]. High *ABCA1* expression was significantly associated with reduced PFS when only HGSOC was included in the analysis [Table 1B]. High A*BCA1* and *ABCC2* mRNA were significantly associated with reduced PPS in all ovarian cancer subtypes [Table 1A] and HGSOC [Table 1B]. High *ABCB3* mRNA expression was significantly associated with increased PPS in all ovarian cancer subtypes [Table 1A] and HGSOC [Table 1B]. Only high *ABCB3* expression was significantly associated with increased OS in patients with HGSOC [Table 1B].

**Table 1:** Relationship between ABC transporter mRNA expression and ovarian cancer outcome using Kaplan-Meir plotter

ABC transporter	Progression-free survival			Post-progression-free survival			Overall survival		
	HR	95%CI	*P* value	HR	95%CI	*P* value	HR	95%CI	*P* value
**(A) All ovarian cancers**									
**ABCA1**	**1.20 (*n* = 1435)**	**(1.05-1.38)**	**0.009**	**1.22 (*n* = 782)**	**(1.02-1.46)**	**0.028**	1.08 (*n* = 1656)	(0.94-1.24)	0.27
ABCB1	1.12 (*n* = 1435)	(0.98-1.28)	0.088	0.86 (*n* = 782)	(0.7-1.05)	0.13	1.07 (*n* = 1656)	(0.94-1.22)	0.31
**ABCB3**	**1.67 (*n* = 1435)**	**(1.36-2.05)**	**< 0.0001**	**0.72 (*n* = 782)**	**(0.56-0.92)**	**0.008**	0.85 (*n* = 1656)	(0.68-1.07)	0.17
**ABCC2**	**0.85 (*n* = 1435)**	**(0.75-0.96)**	**0.011**	**1.23 (*n* = 782)**	**(1.02-1.48)**	**0.029**	1.13 (*n* = 1656)	(0.99-1.30)	0.075
**ABCG2**	**0.87 (*n* = 1435)**	**(0.77-0.99)**	**0.032**	0.85 (*n* = 782)	(0.72-1.01)	0.059	0.91 (*n* = 1656)	(0.80-1.04)	0.15
**(B) High grade serous ovarian cancers**									
**ABCA1**	**1.20 (*n* = 1029)**	**(1.02-1.4)**	**0.026**	**1.28 (*n* = 698)**	**(1.06-1.55)**	**0.009**	1.11 (*n* = 1144)	(0.94-1.31)	0.21
ABCB1	1.16 (*n* = 1029)	(1.00-1.35)	0.056	1.08 (*n* = 698)	(0.91-1.29)	0.39	0.89 (*n* = 1144)	(0.76-1.05)	0.17
**ABCB3**	**0.73 (*n* = 1029)**	**(0.57-0.94)**	**0.014**	**0.74 (*n* = 698)**	**(0.56-0.97)**	**0.028**	**0.72 (*n* = 1144)**	**(0.57-0.92)**	**0.0076**
ABCC2	1.17 (*n* = 1029)	(0.98-1.38)	0.074	**1.24 (*n* = 698)**	**(1.02-1.52)**	**0.032**	1.15 (*n* = 1144)	(0.96-1.38)	0.12
ABCG2	1.1 (*n* = 1029)	(0.93-1.29)	0.28	0.85 (*n* = 698)	(0.71-1.01)	0.07	0.91 (*n* = 1144)	(0.78-1.06)	0.22

Significant results (*P* < 0.05) are highlighted in bold.

### High ABCA1 and ABCB1 transporter protein expression are associated with reduced overall survival in HGSOC patients

**Figure 1:**
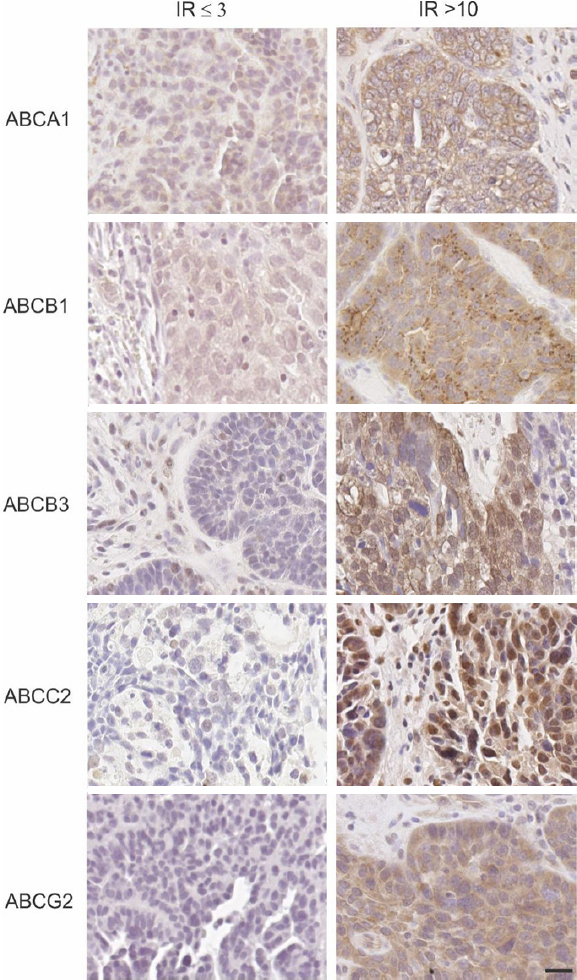
ABC transporter protein expression in high grade serous ovarian cancer patient tissue cohorts. Examples are shown for each transporter [low expression with immunoreactive score (IR) ≤ 3 (left column); high expression with IR > 10 (right column)]. All images are at the same magnification. Scale bar = 20 µm. IR:Immunoreactive score.

**Figure 2:**
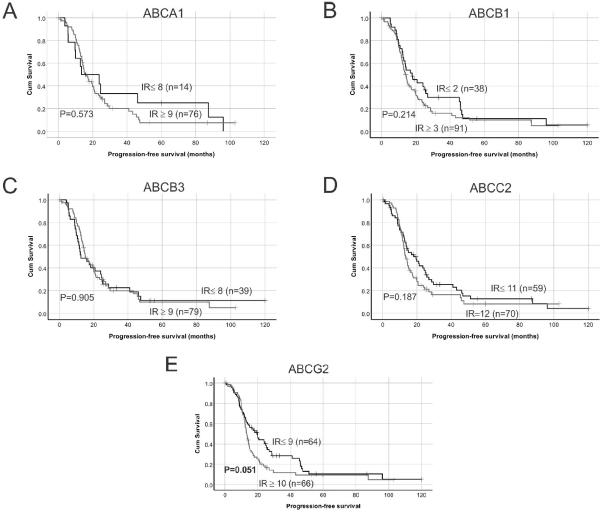
Kaplan-Meier survival analysis showing relationship between ABC transporter protein expression and HGSOC patient progression-free survival (PFS). Median immunoreactive (IR) scores were used as cut-off points to separate samples into groups with low or high ABC transporter protein expression: (A) low ABCA1 expression (IR ≤ 8) *vs.* high ABCA1 expression (IR ≥ 9); (B) low ABCB1 expression (IR ≤ 2) *vs.* high ABCB1 expression (IR ≥ 3); (C) low ABCB3 expression (IR ≤ 8) *vs.* high ABCB3 expression (IR ≥ 9); (D) low ABCC2 expression (IR ≤ 11) *vs.* high ABCC2 expression (IR = 12); and (E) low ABCG2 expression (IR ≤ 9) *vs.* high ABCG2 expression (IR ≥ 10). Data were analyzed using log rank test. IR:Immunoreactive score.

**Figure 3:**
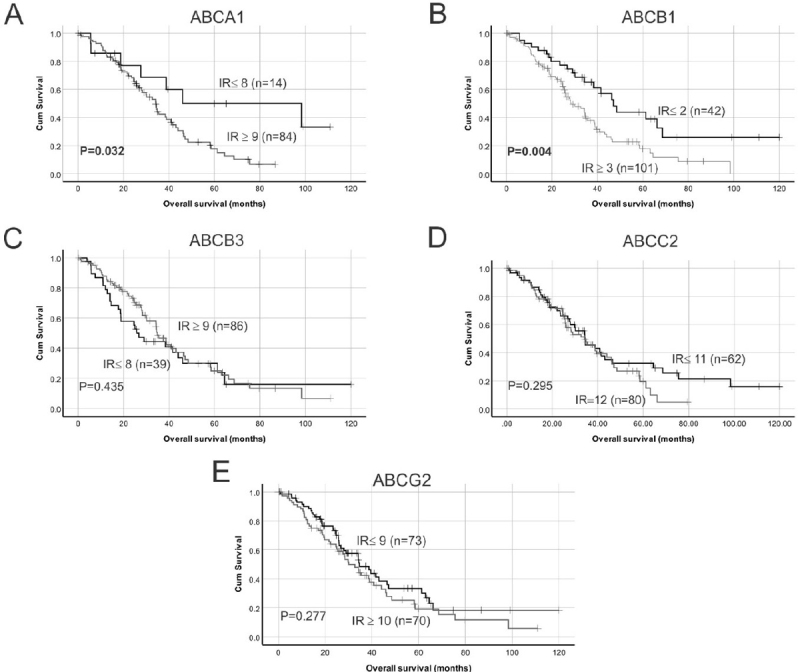
Kaplan-Meier survival analysis showing relationship between ABC transporter protein expression and HGSOC overall survival. Median IR were used as cut-off points to separate samples into groups with low or high ABC transporter protein expression: (A) low ABCA1 expression (IR ≤ 8) *vs.* high ABCA1 expression (IR ≥ 9); (B) low ABCB1 expression (IR ≤ 2) *vs.* high ABCB1 expression (IR ≥ 3); (C) low ABCB3 expression (IR ≤ 8) *vs.* high ABCB3 expression (IR ≥ 9); (D) low ABCC2 expression (IR ≤ 11) *vs.* high ABCC2 expression (IR = 12); and (E) low ABCG2 expression (IR ≤ 9) *vs.* high ABCG2 expression (IR ≥ 10). Data were analyzed using log rank test. IR:Immunoreactive score.

Examples of high and low ABC transporter protein expression in HGSOC patient tissues are shown in Figure 1. Strong staining was observed in positive control in human colon tissues for ABCA1, ABCB1 and ABCB3 and mouse liver tissues for ABCC2 and ABCG2 [Supplementary Figure 2]. Using Kaplan-Meier survival analyses in HGSOC cohort, no significant findings were observed between ABCA1, ABCB1, ABCB3 or ABCC2 protein expression and PFS [Figure 2A-D]. Patients with high ABCG2 IR score (IR ≥ 10) had reduced PFS compared to patients with lower IR score [IR ≤ 9, *P* = 0.051, Figure 2E]. Patients with high ABCA1 (IR score ≥ 9) [Figure 3A, *P* = 0.032] or high ABCB1 (IR score ≥ 3) [Figure 3B, *P* = 0.004] had significantly reduced OS. However, no significant relationship was observed between OS and the expression of the other ABC transporters [Figure 3C-E]. Cox regression analysis confirmed that increased ABCA1 and ABCB1 expression is associated with reduced OS [Table 2A]. Both ABCA1 and ABCB1 expression remained independent predictors of OS when combined in a multivariate analysis [Table 2B]. No relationship was observed between expression of the ABC transporters and PFS using Cox regression analysis (data not shown).

**Table 2:** Cox regression analyses of ABC transporter protein expression in the HGSOC TMA patient cohort

Variable	*n*	Overall survival		
		Relative risk	95%CI	*P* value
**(A) Univariate Cox Regression analyses for progression-free survival and overall survival**				
Age^a^	142	1.35	0.92-1.99	0.172
Tumor stage^b^	143	0.93	0.37-2.30	0.868
Tumor grade^c^	143	0.92	0.54-1.57	0.759
Residual disease^d^	98	2.01	0.95-4.27	0.069
ABCA1^e^	97	**2.47**	**1.05-5.80**	**0.037**
ABCB1^f^	140	**2.04**	**1.24-3.34**	**0.005**
ABCB3^g^	123	0.83	0.52-1.33	0.436
ABCC2^h^	139	1.26	0.82-1.95	0.296
ABCG2^i^	140	1.29	0.85-1.96	0.228
**(B) Multivariate Cox Regression analyses for overall survival (*n* = 97)**				
ABCA1^e^		**2.33**	**1.19-7.89**	**0.050**
ABCB1^f^		**1.93**	**0.25-0.89**	**0.033**

NOTE: *P* values highlighted in bold indicate *P* < 0.05; ^a^age as a dichotomous variable, cut-off point < 55 *vs.* ≥ 55; ^b^tumor stage (FIGO stage II + III *vs.* FIGO stage IV); ^c^tumors grade (moderate *vs.* poor); ^d^ residual disease status (negative *vs.* positive); ^e^ABCA1 (IR) in cancer cells as a dichotomous variable, cut-off point ≤ 8 *vs.* ≥ 9 (only 5/7 TMA slides available for the immunostaining); ^f^ABCB1 (IR) in cancer cells as a dichotomous variable, cut-off point ≤ 2 *vs.* ≥ 3; ^g^ABCB3 (IR) in cancer cells as a dichotomous variable, cut-off point ≤ 8 *vs.* ≥ 9; ^h^ABCC2 (IR) in cancer cells as a dichotomous variable, cut-off point ≤ 11 *vs.* ≥ 12; ^i^ABCG2 (IR) in cancer cells as a dichotomous variable, cut-off point ≤ 9 *vs.* ≥ 10; IR: immunoreactive score.

### ABCA1 expression is increased in serous ovarian cancer cells with acquired chemotherapy resistance

**Figure 4:**
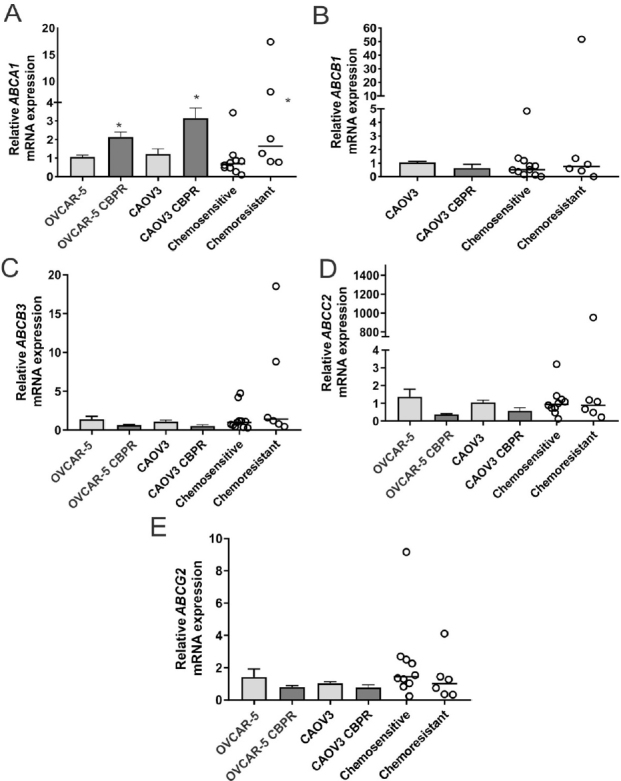
*ABCA1* mRNA expression is increased in ovarian cancer cells with acquired carboplatin resistance. (A) *ABCA1*; (B) *ABCB1*; (C) *ABCB3*; (D) *ABCC2*; and (E) *ABCG2* expression in parental (OVCAR-5 and CaOV3) and carboplatin resistant ovarian cancer cell lines (OVCAR-5 CBPR and CaOV3 CBPR). For the cell line, the columns represent the mean fold changes ± SEM from 12-20 RNA samples from 4-6 independent experiments. *P < 0.05, unpaired *t* test. Data for the primary HGSOC cells from patients with chemosensitive at diagnosis (*n* = 9) or acquired chemoresistance following relapse (*n* = 6) are expressed as the median fold change from 3-6 RNA samples from two independent experiments. *P = 0.026, Mann-Whitney *U* test. *ABCB1* was not detectable in OVCAR-5 cells.

**Figure 5:**
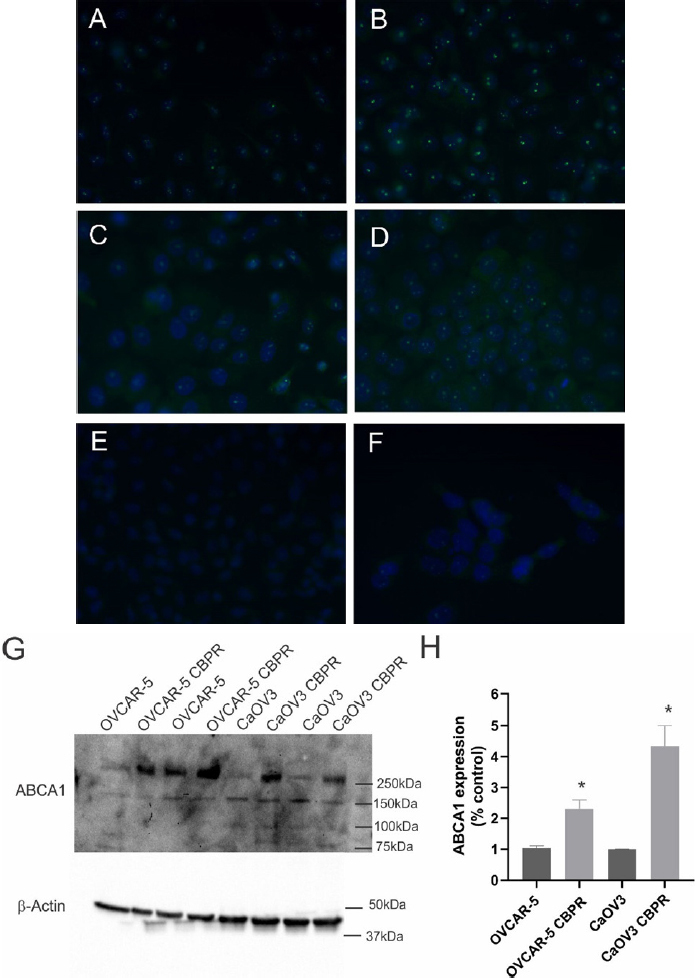
ABCA1 protein expression is upregulated in ovarian cancer cells with acquired carboplatin resistance. ABCA1 expression in OVCAR-5 (A) and CaOV3 (C) and carboplatin resistant OVCAR-5 CBPR (B) and CaOV3 CBPR (D) by immunocytochemistry using rabbit polyclonal ABCA1 antibody (1/100, NB400-105, Novus Biological). (E) OVCAR-5 cells with Rabbit IgG and (F) CaOV3 cells with Rabbit IgG. (G) Protein extracts from OVCAR-5 (~30 µg) and CaOV3 cell lines (~60 µg) were electrophoresed and immunoblotted with rabbit polyclonal ABCA1 antibody (1/1000, NB400-105, Novus Biological), and β-actin (1/2000, Abcam) was used as a loading control. A major band was detected at ~250 kDa, which is the predicted size for ABCA1. (H) Quantitation of ABCA1 Western blots. Data are from 2-4 independent experiments. Statistical significance was determined using the Student’s *t*-test, *P < 0.05.

*ABCA1* mRNA expression was significantly increased in OVCAR-5 CBPR (~2-fold, *P* = 0.017) and CaOV3 CBPR (~3-fold, *P* = 0.007) ovarian cancer cell lines, compared to parental OVCAR-5 and CaOV3 cell lines [Figure 4A]. *ABCA1* mRNA levels were significantly increased in primary HGSOC cells obtained from patients with acquired chemotherapy resistance at relapse compared to patients with chemosensitive disease at diagnosis [Figure 4A, ~2-fold increase, *P* = 0.042]. No significant differences in mRNA expression for *ABCB1*, *ABCB3*, *ABCC2* or *ABCG2* were observed between carboplatin resistant ovarian cancer cell lines (OVCAR-5 CBPR and CaOV3 CBPR) and parental cells (OVCAR-5 and CaOV3) [Figure 4B-E]. Similarly, no difference in mRNA expression for *ABCB1, ABCB3, ABCC2* or *ABCG2* was observed between primary HGSOC cells from patients with chemosensitive disease at diagnosis and patients with acquired chemotherapy resistance at relapse [Figure 4B-E]. Increased ABCA1 protein expression was observed in both OVCAR-5 CBPR and CaOV3 CBPR compared to the parental cells by immunocytochemistry [Figure 5A-D]. ABCA1 was localized to both the cytoplasm and nucleus of the cells. No staining was observed in OVCAR-5 [Figure 5E] or CaOV3 cells [Figure 5F] incubated with rabbit IgG instead of ABCA1 antibody. Increased ABCA1 protein expression in OVCAR-5 CBPR and CaOV3 CBPR cells compared to the parental cells was confirmed by Western blotting [Figure 5G and H].

### Apabetalone reduces ABCA1 expression and overcomes carboplatin resistance

**Figure 6:**
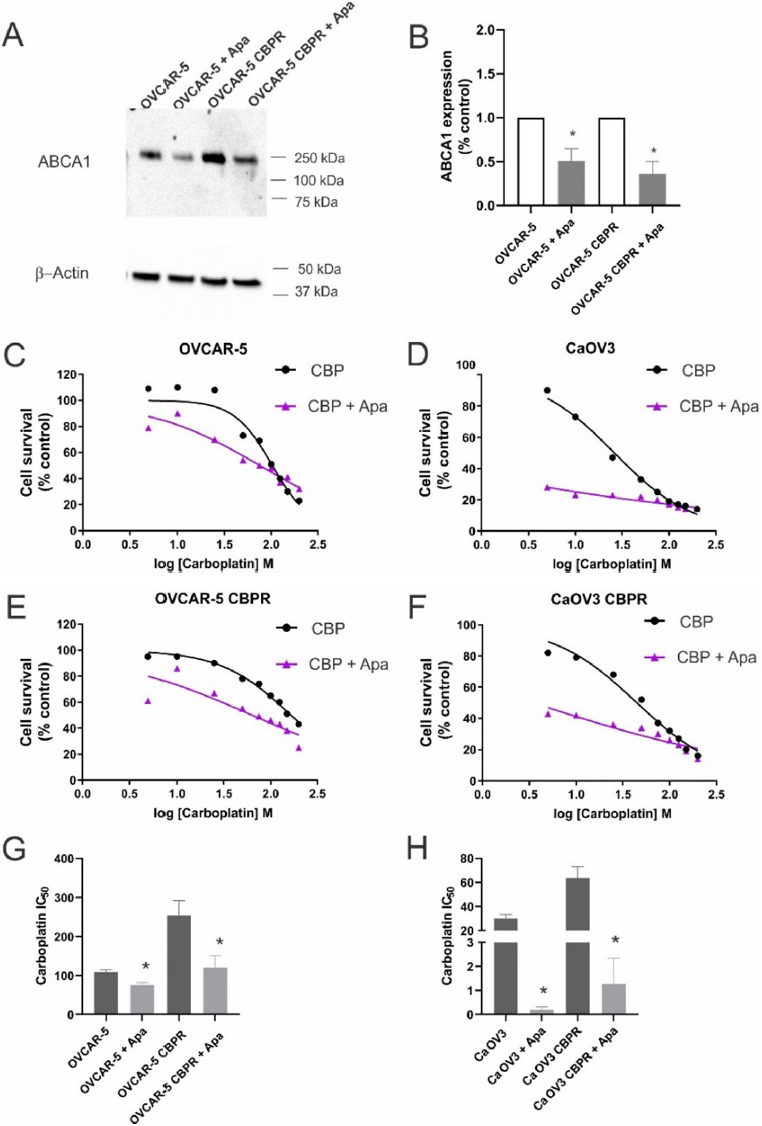
Effects of apabetalone on ABCA1 expression and carboplatin response in ovarian cancer cells. (A) Apabetalone treatment reduced expression of ABCA1 in OVCAR-5 and OVCAR-5 CBPR cell lines. Cells were treated for 72 h with control medium (DMSO) or apabetalone (80 µM). Protein extracts from OVCAR-5 (~30 µg) were electrophoresed and immunoblotted with rabbit polyclonal ABCA1 antibody (1/1000, NB400-105, Novus Biological) and β-actin (1/2000, Abcam) was used as a loading control. (B) Quantification of Western blot. OVCAR-5 (C), CaOV3 (D), OVCAR-5 CBPR (E) and CaOV3 CBPR (F) cell survival following treatment with carboplatin (CBP) alone (0-200 µM, black line) and in combination with apabetalone (Apa, 80 µM, purple line). (G) Carboplatin IC_50_ for OVCAR-5 and OVCAR-5 CBPR cells ± apabetalone. Data are mean ± SEM from three independent experiments. *P < 0.05, Student’s *t*-test. (H) Carboplatin IC_50_ for CaOV3 and CaOV3 CBPR cells ± apabetalone. Data are mean ± SEM from three independent experiments. *P* < 0.05, Student’s *t*-test.

We investigated whether an inhibitor of ABCA1, apabetalone^[10]^, could decrease ABCA1 expression in ovarian cancer cell lines and increase their sensitivity to carboplatin. OVCAR-5 cells were treated for 48 h with increasing concentrations of apabetalone (0-80 µM). Apabetalone treatment (80 µM) for 48 h reduced ABCA1 protein expression in OVCAR-5 cells [Supplement Figure 3A and B]. Cell survival of OVCAR-5 cells was inhibited following 72 h treatment with 40 and 80 µm apabetalone [Supplement Figure 3C]. We confirmed that 80 µm apabetalone treatment significantly inhibited ABCA1 protein expression in OVCAR-5 and matching carboplatin resistant cells OVCAR-5 CBPR cells [Figure 6A and B]. Co-treatment with apabetalone (80 µM) increased the sensitivity to carboplatin for OVCAR-5 [Figure 6C] and CaOV3 [Figure 6D] cells and the matching carboplatin resistant cells [Figure 6E and F]. The carboplatin IC_50_ was significantly reduced when parental or carboplatin resistant cell lines were co-treated with carboplatin and apabetalone [Figure 6G and H]. The combination of apabetalone and carboplatin was synergistic in carboplatin-resistant cells (combination index < 1.0) but not in carboplatin-sensitive cells [Supplementary Figure 4].

## Discussion

Clinical trials evaluating three generations of ABC inhibitors (e.g., nicardipine, biricodar and sulindac) targeting specific transporters (e.g., ABCB1 and ABCG2) have failed because of immunosuppression, nephrotoxicity and interaction with chemotherapeutics^[7,45-47]^. The trials did not select patients by ABC transporter expression, and clinical trials targeted ABCB1 and ABCG2 (with only a few targeting ABCC2), but other ABC transporters also likely to play important roles in ovarian cancer progression and chemotherapy resistance^[7]^. Our results highlight that increased expression of ABCA1 is associated with development of acquired chemotherapy resistance and poor patient outcome.

ABCA1 is a transmembrane protein responsible for reverse cholesterol transport from inside cells into the blood^[48]^ and interacts with apolipoprotein A1 to bind cholesterol and synthesize high-density lipoproteins^[49]^. Both the overexpression and the downregulation of ABCA1 have been associated with tumorigenesis including ovarian cancer^[11,12,50-55]^. In this study, we showed that high *ABCA1* mRNA was significantly associated with both reduced PFS and PPS but not OS, while high protein ABCA1 expression was associated with reduced OS. Together, our findings are in agreement with a previous study showing that high *ABCA1* mRNA expression was associated with reduced PFS and OS in ovarian cancer patients from The Cancer Genome Atlas (TCGA, *n* = 407)^[11]^ The same study also found reduced PFS and OS in HGSOC patients with high ABCA1 protein expression (*n* = 91)^[11]^. However, another study observed that low ABCA1 protein expression was associated with reduced PFS (*n* = 55, *P* = 0.038)^[51]^. This disparate finding may be due to the smaller cohort size, including different ovarian cancer subtypes and a mixture of both low- and high-grade disease, varied chemotherapy treatment, different methods of assessment and the use different antibodies to detect ABCA1.

Increased ABCA1 mRNA and protein expression were observed in both carboplatin-resistant OVCAR-5 and CaOV3 cell lines compared to the parental cells and correlated with the 2-3-fold increase in carboplatin IC_50_. A similar fold increase in *ABCA1* expression was observed between primary cells from patients with chemosensitive disease and acquired chemotherapy resistance. The first evidence for the role of ABCA1 in platinum chemotherapy resistance comes from a study that demonstrated increased *ABCA1* mRNA expression in a cisplatin resistant epidermoid carcinoma cell line (KCP-4)^[56]^. Lentiviral knockdown of *ABCA1* in KCP-4 cells resulted in the re-sensitization to cisplatin^[56]^. A more recent study investigating drug-resistant tumor cell phenotypes in the ascitic fluid of epithelial ovarian cancer patients identified that a population of cells that were EpCAM^+^CD45^+^ were more resistant compared to EpCAM^+^ tumor cells and overexpressed *ABCA1*^[57]^. A limitation of our study was not performing knockdown of *ABCA1* to demonstrate reversal of carboplatin resistance.

This study found that increased ABCB1 protein expression was associated with reduced OS in HGSOC. It is not clear why *ABCB1* mRNA and protein expression results are contrasting, but discord may be due increased mRNA turnover^[58]^ or mRNA instability^[59]^ compared to protein half-life^[60]^. Our findings are in agreement with previous HGSOC studies demonstrating a significant relationship between ABCB1 protein expression and reduced OS (*n* = 60, *P* = 0.015^[14]^; *n* = 52, *P* < 0.0005^[13]^). A more recent study found a significant relationship between high *ABCB1* expression and reduced PFS (*n* = 143, *P* = 0.003) in patients with HGSOC^[61]^. Although increased ABCB1 mRNA and protein expression have been shown in chemoresistant ovarian cancer cell lines^[62-66]^, our study found no significant relationship between *ABCB1* expression and carboplatin-resistance. ABCB1 is expressed in ovarian tumors which have been treated with paclitaxel, but not in chemonaive cancers or cancers exposed to other chemotherapy drugs such as cisplatin that are not substrates for ABCB1^[7,67-69]^.

We found high *ABCB3* mRNA expression was significantly associated with increased PFS, PPS and OS when only HGSOC patients were included in the analysis. In contrast, no significant relationship was observed between ABCB3 protein expression levels and patient outcome. This may be due to reduced mRNA turnover^[58]^ or increased mRNA stability^[59]^ compared to protein half-life^[60]^. Our findings agree with a large cohort study (*n* = 232 EOC including *n* = 128 serous OC) that observed no association between ABCB3 protein expression and clinical outcome^[21]^.

ABCB3 was upregulated in breast cancers following treatment with neo-adjuvant chemotherapy and increased in recurrent ovarian cancers^[19,70]^. Our previous study also showed higher ABCB3 protein levels in ovarian cancer tissues after neoadjuvant carboplatin treatment and after recurrence compared with tissues from untreated ovarian cancers^[7]^. Although these studies suggested a link between ABCB3 expression and chemotherapy resistance, our study did not find a relationship between ABCB3 expression and acquired chemotherapy resistance.

ABCC2 mRNA or protein levels have been shown to be associated with ovarian cancer outcome^[19,22,23]^, but several other studies did not find a relationship with patient outcome^[24,25,71,72]^. In our analysis, we found that high *ABCC2* mRNA expression was significantly associated with reduced PPS in all ovarian cancer and HGSOC, but we did not find any relationship between patient outcome and ABCC2 protein expression. The reasons for the inconsistency are unknown, but it may due to the use of small patient cohorts, inclusion of different ovarian cancer subtypes and the use of different primary antibodies. Another explanation for the discrepancy may be due to the ABCC2 localization within the cell. Surowiak *et al.*^[22]^ found that higher nuclear ABCC2 levels both before and after chemotherapy were associated with cisplatin resistance and shorter survival time. A limitation of our study was not assessing nuclear expression of ABCC2. Although previous studies found ABCC2 mRNA and protein expression to be increased by carboplatin treatment and following recurrence^[7,19,42]^, in this study, we did not find a relationship between ABCC2 expression and acquired chemotherapy resistance. This observation is consistent with previous studies that did not observe a relationship between platinum response and ABCC2 mRNA or protein expression^[25,71-74]^.

High ABCG2 protein levels but not *ABCG2* mRNA expression were significantly associated with reduced PFS in HGSOC patient tissues in this study. It is not clear why *ABCG2* mRNA and protein expression results are contrasting, but discord may be due to increased mRNA turnover^[58]^ or mRNA instability^[59]^ compared to protein half-life^[60]^. Several studies have investigated the relationship between *ABCG2* genotype variants with ovarian cancer outcome. One study found no relationship between a variant *ABCG2* and clinical outcome in a large mixed subtype cohort of ovarian cancer patients (*n* = 914)^[75]^ while a more recent study found that a *ABCG2* variant (C421A) previously linked with enhanced protein degradation and drug sensitivity^[76]^ was associated with longer PFS in patients with advanced stage epithelial ovarian cancer treated with platinum + paclitaxel-based chemotherapy^[77]^.

ABCG2 is a marker of ovarian cancer stem cells and highly expressed in ovarian cancer spheroids which have higher chemoresistance to cisplatin or paclitaxel^[27,78]^. However, we did not observe a significant relationship between ABCG2 expression and acquired chemoresistance, which is in agreement with another study that did not observe any difference in ABCG2 expression in A2780 ovarian cancer cell treated with cisplatin^[79]^. However, a significant overexpression of ABCG2 mRNA and protein was observed in topotecan-resistant ovarian cancer cell lines^[80,81]^. Another study challenged ovarian cancer cell lines with six different chemotherapies and found increased *ABCG2* mRNA expression in vincristine- (*P* < 0.01) and topotecan-resistant (*P* < 0.001) cell lines^[63]^. Together, the data indicate that ABCG2 is not increased following carboplatin chemotherapy treatment but may play a role in chemoresistance to other chemotherapy drugs including vincristine and topotecan.

Apabetalone has recently been shown to inhibit ABCA1 protein expression in Caco-2 colon cancer cells and inhibit tumor promoting behavior including proliferation, migration, invasion and reverse the EMT phenotype observed in ABCA1 overexpressing Caco-2 cells^[10]^. Importantly, treatment with apabetalone signiﬁcantly decreased cholesterol transport in both control cells and ABCA1 overexpressing Caco-2 cells^[10]^. In our study, we found that apabetalone treatment also reduced ABCA1 protein expression and increased the sensitivity of ovarian cancer cell lines to carboplatin. Apabetalone is a bromodomain and extra-terminal (BET) inhibitor that has recently been investigated for the treatment of atherosclerosis in clinical trials^[82,83]^. The concentration of apabetalone used in our experiments was similar to that used in *in vitro* studies using colon cancer cell lines^[10]^ and hepatocytes^[84]^. Apabetalone appears to be well tolerated in humans (50-150 mg twice/day^[82,85]^) and mice (150 mg/kg b.i.d for 14 weeks)^[86]^. Pharmacokinetic studies in humans administering 100 mg apabetalone have observed a peak plasma concentration of 360 ng/mL (~1 µM) after 3.5 h treatment^[87]^, which is considerably lower than the concentration used in our study. Further experiments need to be performed to examine the feasibility of using apabetalone and the doses required to reduce ABCA1 expression in *in vivo* ovarian cancer models.

In conclusion, we provided evidence for the role of ABCA1 in ovarian cancer acquired carboplatin resistance and progression. Our study found that high ABCA1 mRNA and protein expression was significantly associated with poor clinical outcome and increased in ovarian cancer cell lines and primary serous ovarian cancer cells following acquired chemotherapy resistance. Developing strategies to inhibit ABCA1 expression has potential to overcome chemotherapy resistance and improve ovarian cancer survival.

## Declarations

### Copyright

© The Author(s) 2021.
