# Supplementary material for: ABCA1 is associated with the development of acquired chemotherapy resistance and predicts poor ovarian cancer outcome
Source: Cancer Drug Resist. 2021 Jun 19;4(2):485–502. doi: 10.20517/cdr.2020.107 (PMC9019266; doi:10.20517/cdr.2020.107)
Supplement: Supplementary file 1 [file cdr-4-485-SupplementaryMaterials.pdf]

**Supplementary Table 1.** Clinicopathological characteristics of serous ovarian cancer cohort

| **High grade serous ovarian carcinomas (n=147)** | | |
|---|---|---|
| Age at Diagnosis (years) | Median (range) | 61 (24-87) |
| Histological Grade | Grade 2<br>Grade 3 | 22<br>125 |
| FIGO stage | Stage II<br>Stage III<br>Stage IV | 3<br>136<br>8 |
| Residual disease after surgery | No<br>Yes<br>Unknown | 20<br>81<br>46 |
| ABCA1 immunoreactive score | Median (range)<br>Patient number<br>unstained/lost cores | 9 (4-12)<br>n=99<br>48 |
| ABCB1 immunoreactive score | Median (range)<br>Patient number<br>Lost cores | 3 (0-12)<br>n=144<br>3 |
| ABCB3 immunoreactive score | Median (range)<br>Patient number<br>Lost cores | 9 (0-12)<br>n=126<br>21 |
| ABCC2 immunoreactive score | Median (range)<br>Patient number<br>Lost cores | 12 (0-12)<br>n=143<br>4 |
| ABCG2 immunoreactive score | Median (range)<br>Patient number<br>Lost cores | 12 (0-12)<br>n=144<br>3 |
| Treatment | Cisplatin/cyclophosphamide<br>Carboplatin/paclitaxel<br>Carboplatin alone<br>Other chemotherapy<br>No chemotherapy<br>Unknown | 53<br>56<br>11<br>11<br>7<br>9 |
| Recurrence | No<br>Yes<br>Unknown | 24<br>108<br>15 |
| Cause of Death | Ovarian cancer<br>Other cause<br>Alive<br>Lost to follow-up | 92<br>14<br>40<br>1 |
| Residual Disease | Yes<br>No<br>Unknown | 81<br>20<br>46 |

**Supplementary Table 2:** Summary of clinical and pathological characteristics of the primary ovarian cancer cells established from patient ascites

| Patient | Age at Diagnosis (years) | Stage at Diagnosis | Diagnosis | 1st line treatment | Chemosensitive |
|---|---|---|---|---|---|
| 1 | 46 | IIIC | Serous papillary carcinoma of the ovary | Carboplatin & Paclitaxel | Yes |
| 2 | 66 | IV | Recurrent ovarian carcinoma | Carboplatin & Paclitaxel | Yes |
| 3 | 66 | IIIC | Serous carcinoma of ovary/peritoneum | Carboplatin & Paclitaxel | Yes |
| 4 | 60 | IIIC | Serous papillary carcinoma of the ovary | Carboplatin & Paclitaxel | Yes |
| 5 | 80 | IIIC | Serous papillary carcinoma of the ovary | Carboplatin | Yes |
| 6 | 72 | IIIC | Serous papillary carcinoma of the ovary | Carboplatin & Paclitaxel | Yes |
| 7 | 61 | IIIA | Papillary serous carcinoma of the ovary | Carboplatin & Paclitaxel | Yes |
| 8 | 58 | IIIC | Serous papillary carcinoma of the ovary | Carboplatin & Paclitaxel | Yes |
| 9 | 66 | IIIC | Serous papillary carcinoma of the ovary | Carboplatin & Paclitaxel | Yes |
| 10 | 85 | IIIC | Serous papillary carcinoma of the ovary | Carboplatin / Paclitaxel + Caelyx | No |
| 11 | 47 | IIIC | Recurrent serous carcinoma of the ovary | Carboplatin & Paclitaxel | No |
| 12 | 69 | IIIA | Recurrent serous ovarian cancer | Carboplatin & Paclitaxel | No |
| 13 | 59 | IA | Recurrent serous tubal | No chemotherapy | No |
| 14 | 43 | IIC | Recurrent serous peritoneal carcinoma | Carboplatin & Paclitaxel | No |
| 15 | 57 | - | Recurrent serous carcinoma | Carboplatin & Paclitaxel | No |

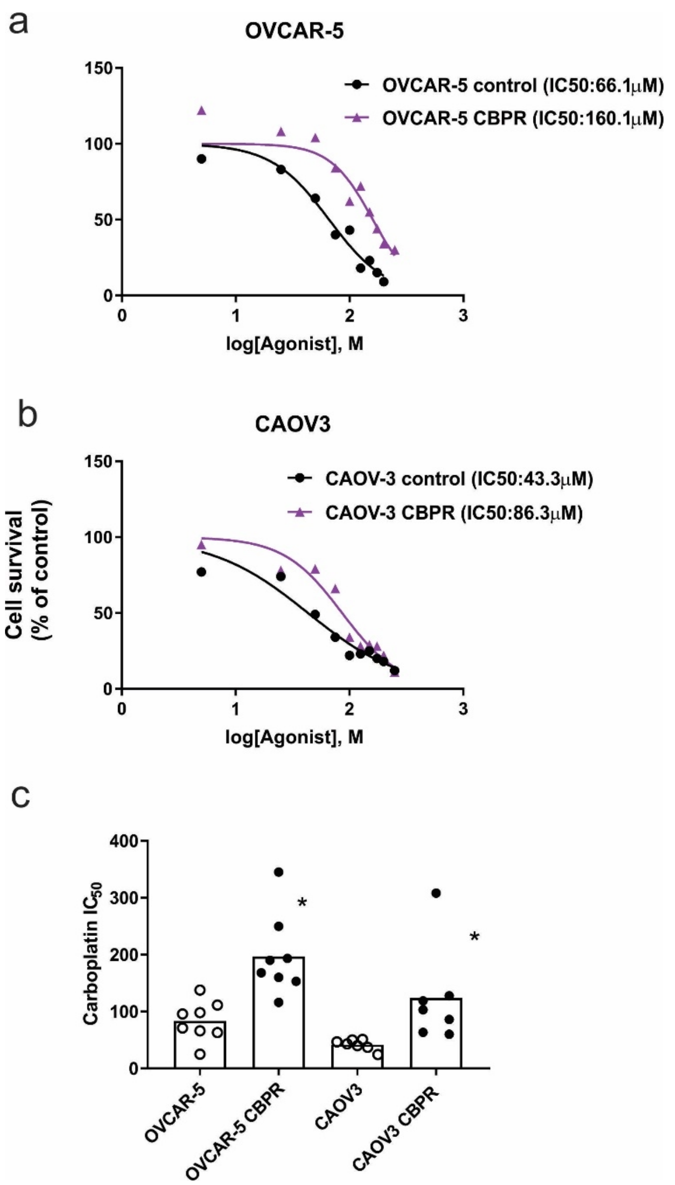


**Supplementary Figure 1**. Effect of carboplatin on parental and carboplatin resistant ovarian cancer cells. A. Carboplatin dose (1-200µM) response curves in OVCAR-5 and OVCAR-5 CBPR cell lines. B. Carboplatin dose (1-200µM) response curves in CAOV3 and CAOV3 CBPR cell lines. C. Carboplatin $IC_{50}$ in parental (OVCAR-5 & CAOV3) and carboplatin resistant cell lines (OVCAR-5 CBPR & CAOV3 CBPR). Each data point is the average $IC_{50}$ from triplicate determinations (n=6-7 independent experiments). * $P<0.05$, Student T test.

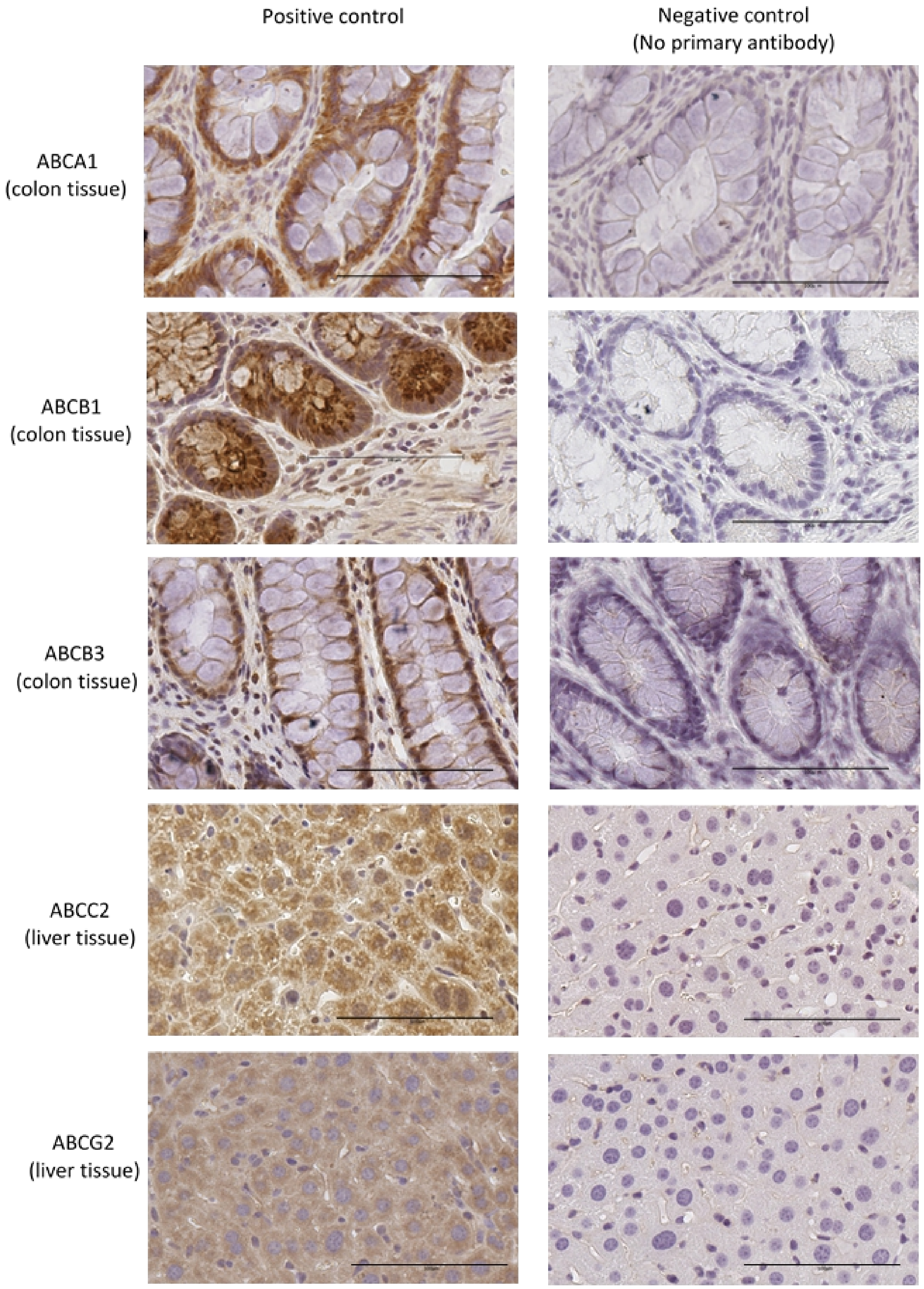


]

**Supplementary Figure 2.** ABC transporter protein expression in human colon and mouse liver tissues. Expression of ABCA1, ABCB1 and ABCB3 in human colon tissue. Expression for ABCC2 and ABCG2 in mouse liver tissue (left column). Matching negative controls are shown with no primary antibody (right column). All images are at the same magnification. Scale bar= 100μm

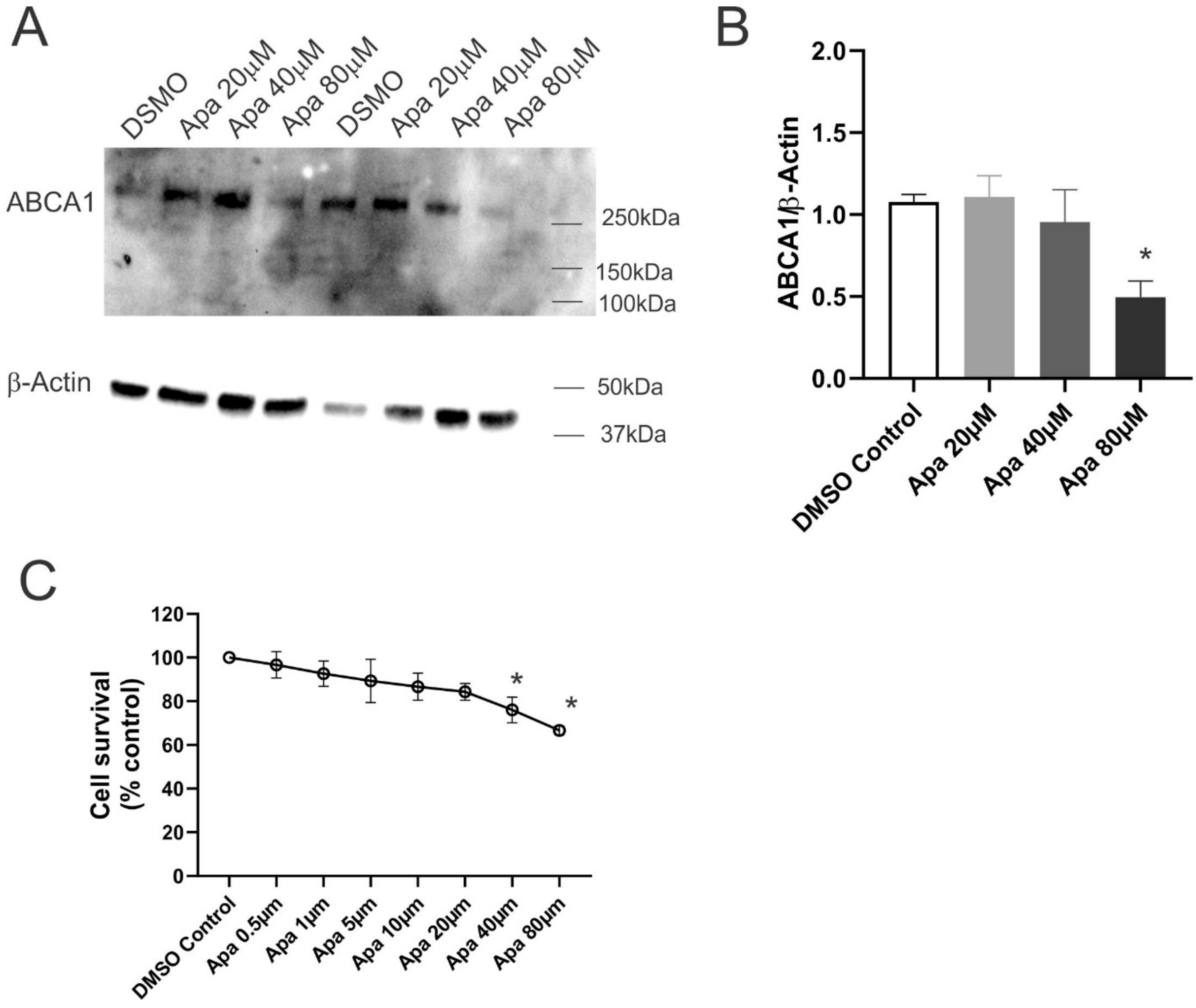


**Supplementary Figure 3.** Effect of apabetalone treatment on OVCAR-5 cells. A. ABCA1 Western blot with increasing concentration of apabetalone (0-80μM). Protein extracts from OVCAR-5 (~30μg) were electrophoresed and immunoblotted with rabbit polyclonal ABCA1 antibody (1/1000, NB400-105, Novus Biological) and β-actin antibody (1/2000, Abcam) was used as a loading control. B. Quantitation of ABCA1 western blot. Data from 2-3 independent experiments. P <0.05, One-Way ANOVA. C. Effect of apabetalone on OVCAR-5 cell survival. Cells were treated with increasing concentrations of apabetalone (0-80μM). Data from mean triplicate determinations from 3 independent experiments. * P<0.05, One way ANOVA.

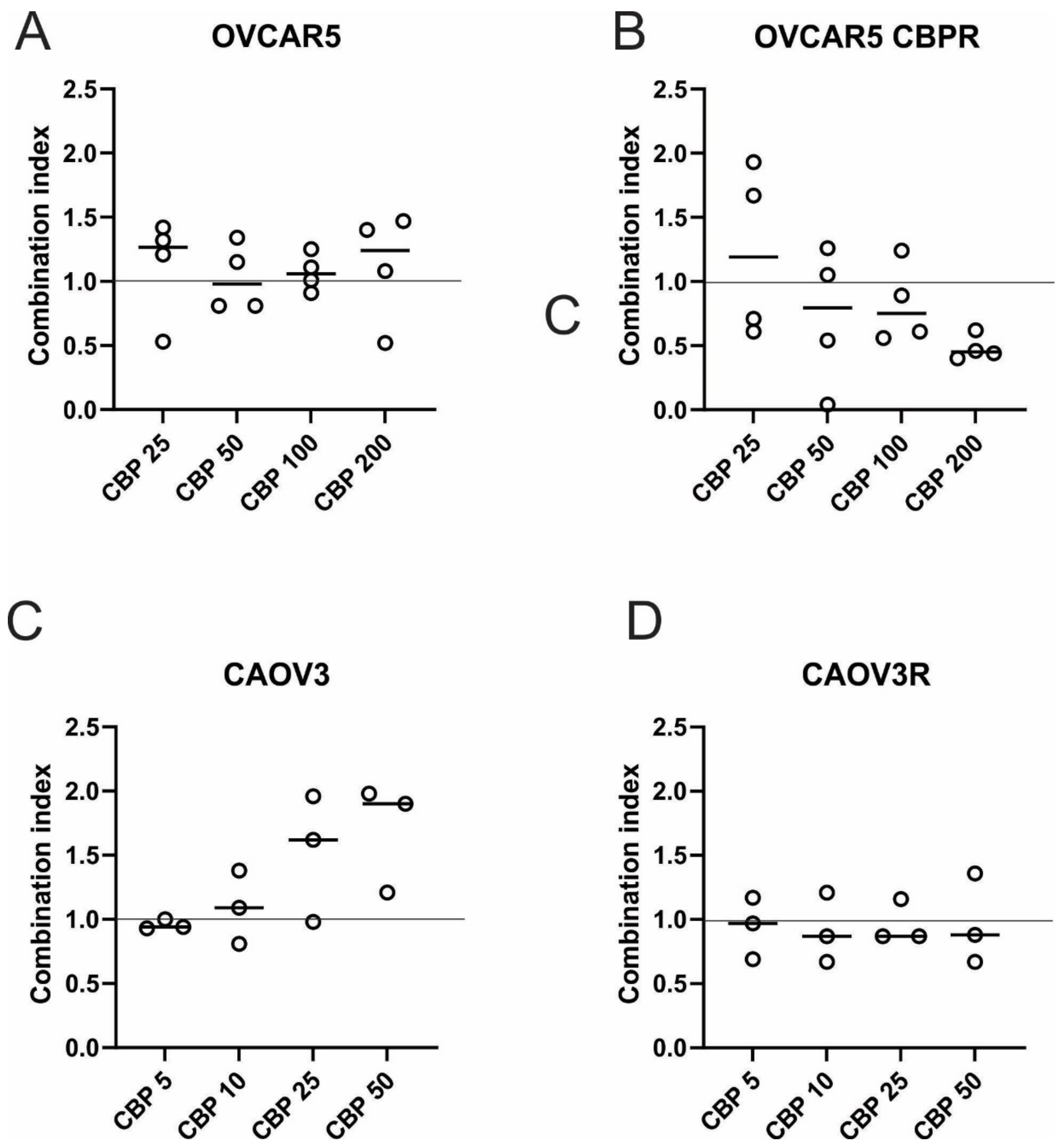


**Supplementary Figure 4.** Combination index for apabetalone and carboplatin treatment A) OVCAR-5, B, OVCAR-5 CBPR, C) CAOV3 and D) CAOV3 CBPR cell lines. Combination index were calculated using the Chou-Talalay method [34] using CompuSyn software (ComboSyn, New Jersey, USA) for apabetalone (80µM) with carboplatin (CBP) 25-200µM (A & B) and apabetalone (80µM) with 5-50µM CBP (C & D). Each data point is the average for triplicate determinations. The bar shows the median combination index from three independent experiments. Drug interactions were synergistic (combination index <1) for OVCAR-5 CBPR (B, CBP 50-200) and CAOV3 CBPR (D, CBP 10-50) cell lines .
